# Anticoagulant Treatment of a Thrombosed Giant Portal Vein Aneurysm

**DOI:** 10.5334/jbsr.3452

**Published:** 2024-02-01

**Authors:** Jean-François Monville, Nicolas Meurisse, Robert F. Dondelinger

**Affiliations:** 1 Department of Medical Imaging Sankt-Nikolaus Hospital, Eupen, Belgium; 2 Department of Abdominal and Transplant Surgery, University Hospital Sart-Tilman, Liège, Belgium; 3 Department of Medical Imaging University Hospital Sart-Tilman, Liège, Belgium

**Keywords:** Giant Portal Vein Aneurysm, Thrombosis and Anticoagulation

## Abstract

*Teaching point:* Anticoagulation is advised in thrombosed portal vein aneurysm (PVA) without portal hypertension.

A 69-year-old female was admitted for moderate epigastric pain lasting for several days. The epigastrium was slightly indurated. The medical history was unrelated. The serum C-reactive protein (CRP) level was 24 mg/L. Abdominal CT obtained 10 years earlier showed a patent portal vein aneurysm (PVA) of 64 mm in diameter with thin margins (Figure 1). Abdominal CT at admission confirmed PVA with a diameter of 79 mm. Native luminal densities of 73 HU corresponded to acute thrombosis (Figure 2). The occlusive thrombus extended into the portal vein branches and into the splenic and superior mesenteric veins. Minimal periportal oedema was present. No thrombophilia was found. Medical treatment included two daily injections of Clexane 60 mg, followed by apixaban 5 mg twice a day for 6 months. Abdominal CT obtained after a follow-up of 14 months showed a reduction of the thrombosed PV diameter to 33 mm, regression of the spleno-mesaraic vein thrombus, recanalisation of the left PV branch and the formation of an extensive periportal cavernoma involving pericholecystic veins (Figure 3). No sign of portal hypertension had developed. The patient remained asymptomatic after 20 months.

**Figure 1:**
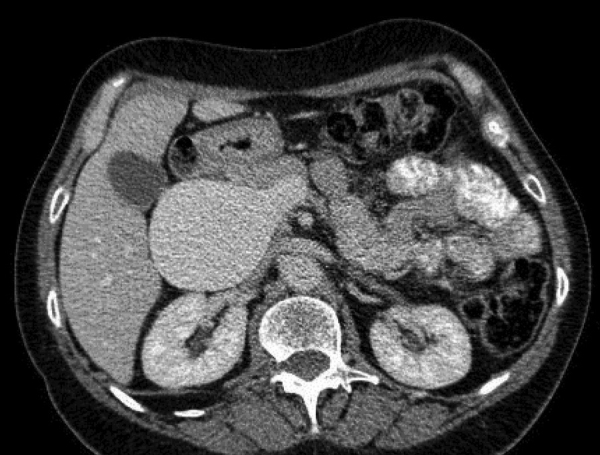
Giant non thrombosed portal vein aneurism.

**Figure 2:**
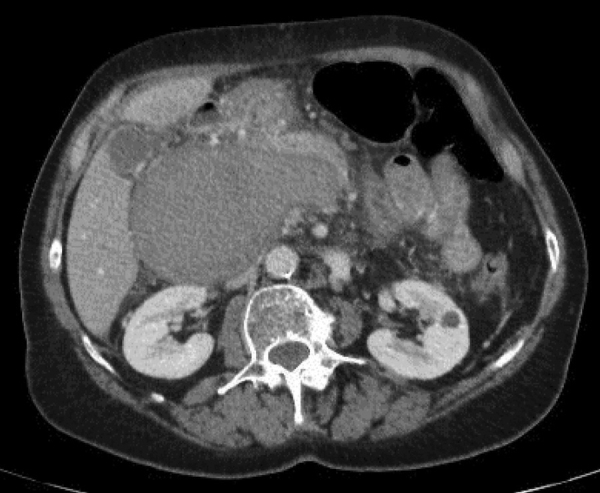
Thrombosed and enlarged portal vein aneurysm.

**Figure 3:**
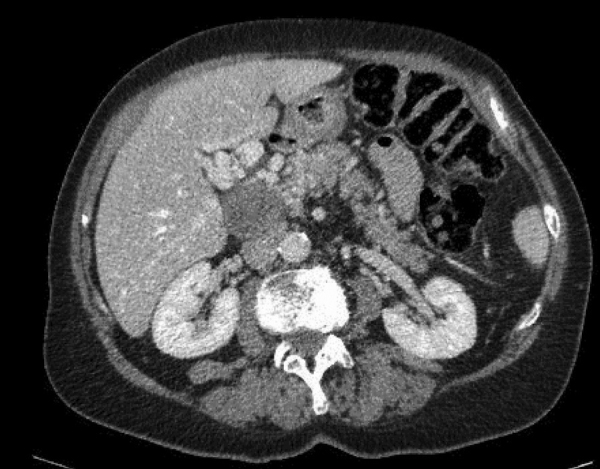
Retracted thrombosed portal vein aneurysm and periportal cavernoma.

PVA is a rare disease; 280 patients being recorded in 2023. A prevalence of 0.43 was reported for a PV dilation exceeding 2 cm. Thrombosis occurred in 19% to 23%. The etiopathogeny of PVA and its natural history are unclear [1]. Incomplete regression of the right primitive distal vitelline vein was thought to be responsible for the congenital variety. Chronic liver disease and portal hypertension were identified as risk factors for acquired PVA. Weakening of the PV wall might be related among other causes to trauma, pancreatitis or tumor. There is no evidence-based management of PVA. Spontaneous regression is exceptional. PVAs with a diameter of up to 3 cm may be clinically observed. Surgical options for large PVAs are shunting procedures in patients with portal hypertension or aneurysmorrhaphy/aneurysmectomy in those without chronic hypertensive liver disease. In past publications, surgery was advocated in 21% of patients, if symptomatic or in case of extended PV thrombosis or risk of rupture. Postoperative mortality was 17.5%. In the handful of published cases who underwent anticoagulant treatment alone, the outcome was favorable. In our patient, smooth clinical evolution and the absence of liver disease or portal hypertension privileged non-operative management. Anticoagulant therapy was able to dissolve the PV thrombus at both ends, but PV patency was not restored. However, the rapid formation of a large cavernoma at the porta hepatis providing for efficient portal drainage prevented portal hypertension to occur and supports the rationale of a conservative strategy in similar cases. Rupture of a thrombosed PVA is extremely rare. Intraluminal catheter-mediated chemical or mechanical thrombus disintegration or stenting, which was occasionally published, was considered non-realistic in our patient in the face of the amount of thrombus and giant aneurysmal size.
